# A system-theoretic approach for image-based infectious plant disease severity estimation

**DOI:** 10.1371/journal.pone.0272002

**Published:** 2022-07-26

**Authors:** David Palma, Franco Blanchini, Pier Luca Montessoro

**Affiliations:** 1 Polytechnic Department of Engineering and Architecture, University of Udine, Udine, Italy; 2 Department of Mathematics, Computer Science and Physics, University of Udine, Udine, Italy; Padjadjaran University: Universitas Padjadjaran, INDONESIA

## Abstract

The demand for high level of safety and superior quality in agricultural products is of prime concern. The introduction of new technologies for supporting crop management allows the efficiency and quality of production to be improved and, at the same time, reduces the environmental impact. Common strategies to disease control are mainly oriented on spraying pesticides uniformly over cropping areas at different times during the growth cycle. Even though these methodologies can be effective, they present a negative impact in ecological and economic terms, introducing new pests and elevating resistance of the pathogens. Therefore, consideration for new automatic and accurate along with inexpensive and efficient techniques for the detection and severity estimation of pathogenic diseases before proper control measures can be suggested is of great realistic significance and may reduce the likelihood of an infection spreading. In this work, we present a novel system-theoretic approach for leaf image-based automatic quantitative assessment of pathogenic disease severity regardless of disease type. The proposed method is based on a highly efficient and noise-rejecting positive non-linear dynamical system that recursively transforms the leaf image until only the symptomatic disease patterns are left. The proposed system does not require any training to automatically discover the discriminative features. The experimental setup allowed to assess the system ability to generalise symptoms detection beyond any previously seen conditions achieving excellent results. The main advantage of the approach relies in the robustness when dealing with low-resolution and noisy images. Indeed, an essential issue related to digital image processing is to effectively reduce noise from an image whilst keeping its features intact. The impact of noise is effectively reduced and does not affect the final result allowing the proposed system to ensure a high accuracy and reliability.

## Introduction

The occurrence of plant diseases cause severe threats to global food security and significant economic losses in yeld and quality as well as affecting agricultural industry all around the world [1].

A plant is said to be healthy when it is able to carry out its physiological functions to the best of its genetic potential. When this ability of the plant is continuously disturbed by either a pathogenic organism or an adverse environmental factor results in an abnormal physiological process that inhibits the normal activities of the plant. This interference with an essential physiological or biochemical system of the plant induces characteristic symptoms or pathological conditions. Initially, the infection is specifically confined to a few plant cells and is not visible. Soon, however, the reaction becomes widespread and affected parts of the plant develop visible or otherwise measurable adverse changes (symptoms), which reflect the amount of disease in the plant [2]. Hence, severity estimation of plant disease is an important procedure to measure the degree of disease and thus can be used to recommend treatment and predict yield, helping to reduce crop losses [3]. Plant diseases can be broadly classified according to the nature of their primary causal agent, either biotic (infectious) or abiotic (non-infectious). The range of phytopathogenic (infectious or parasitic) organisms that attack plants is diverse and includes viruses, mycoplasma, bacteria, fungi, nematodes, protozoa, and parasites, each of which has a unique mode of pathogenicity, whilst non-infectious (non-parasitic) organisms include unfavorable environmental conditions, nutrient deficiencies, disadvantageous relationships between moisture and oxygen, and the presence of toxic chemicals in air or soil [4]. In this study, we consider the case of a specific disease-causing agent due to biotic factors (i.e., those caused by living components such as pathogens).

Traditionally, detection and severity estimation of plant diseases have been mostly performed by human, indeed visual inspection is still the main approach to determine if plants have already been infected presenting various symptoms, which can often be divided in: (i) underdevelopment of tissues or organs (e.g., lack of chlorophyll, leaf malformation), (ii) overdevelopment of tissues or organs, (iii) necrosis of plant parts (leaf spots, leaf blights, wilts), (iv) alternations like mosaic patterns and altered colouration in leaves. The most common way to determine if disease symptoms are present is to seek their presence on leaves, stems, or other plant parts. However, this method relies on experienced professionals performing continuous monitoring of plants, which might be time-consuming, prohibitively expensive as well as prone to considerable risk of error. Plant pathogen detection conventionally relies on molecular assays, including nucleic acid-based and immunological technologies. Various approaches such as fluorescence imaging [5], immunofluorescence techniques [6], thermography [7], chain reactions [8], DNA- or RNA-based affinity biosensor [9], have been often used for quality evaluation of leaves. However, the problems with these techniques lie in the fact that are complicated, time-consuming, and constrained to centralised laboratories [1].

Recent technological developments have allowed useful tools to automatically detect the visually observable patterns (symptoms) that appear on specific parts of a plant, thus helping in the cultivation of healthy plants and improving their quality [10]. Pathologists usually focus on pathogenic diseases appearing particularly on leaves, since on this part of the plant a large amount of information is available allowing an effective diagnosis [11, 12]. Thus, the first step consists of the plant leaf image acquisition which is typically done using consumer-level cameras in a controlled laboratory environment and the format used for the images is RGB quantised with 8 bits. Once the plant leaf images are captured, both image processing and soft computing techniques are applied following a pattern recognition system scheme. However, most estimation methods involve a segmentation step to isolate the symptoms, from which it is possible to extract the features to be properly processed in order to provide a disease severity estimation. Interactive and semiautomatic tools are also available, two of which are considered the most commonly used programs known as Assess [13] and Leaf Doctor App [14] in which the user is asked to interact with the software to achieve the best results to estimate the disease severity. Many of these image-based assessment methods for plant diseases such as those reported in Table 1 rely on the same basic procedure [15, 16]. A comprehensive survey on such a methods for detecting, quantifying, and classifying plant diseases from digital images in the visible spectrum is available in [11].

**Table 1:** Overview of plant disease severity quantification methods.

Reference	Year	Methodology	Culture
Sibiya and Sumbwanyambe [17]	2019	Disease severity estimation system based on the use of colour threshold image segmentation and fuzzy logic inference rules aimed at updating the current algorithm used in the “Leaf Doctor” application [14].	Maize
Wang *et al*. [18]	2017	Image-based plant disease severity estimation system that makes use of a deep learning model fine-tuned by transfer learning to classify the severity into three classes, i.e. early, middle, and final stages, achieving 90.4% accuracy.	Apple
Atoum *et al*. [19]	2016	Computer vision system that extracts multi-scale superpixels, where in each scale a Histogram of Importances feature is used to represent the local characteristics for cercospora leaf spot disease which are fused for learning a regressor that estimates the rating for each plant image.	Sugar beet
Qin *et al*. [20]	2016	Lesion segmentation takes place integrating K-median clustering algorithm and linear discriminant analysis, after that a disease recognition model was built using a support vector machine (SVM) with the most important 45 feature selected from a total of 129 features and achieving 94.74% accuracy.	*Alfalfa*
Barbedo [15]	2014	Image segmentation based on morphological mathematical operations and L*a*b* colour space representation.	Coffee, passion fruit, tomato, peanut, corn
Sekulska-Nalewajko and Goclawski [21]	2011	Disease evaluation using a threshold-based segmentation and a transformation from RGB to HSV colour space on which a Fuzzy c-means algorithm is applied to group the points into healthy and diseased clusters.	Pumpkin, cucumber
Sannakki *et al*. [22]	2011	Disease quantification based on Fuzzy logic. First the images are converted to the L*a*b* colour space, then pixels are grouped into a number of classes through K-means clustering. Finally, a Fuzzy Inference System is used for severity estimation, however, no details are provided [11].	Pomegranate
Bock *et al*. [23]	2009	Semiautomatic approach that first converts the image to the HSI format and then separates the diseased parts from the rest of the scene by manually selecting the threshold to estimate the severity making use of the Assess software [13].	Citrus
Weizheng *et al*. [24]	2008	Lesion detection using a threshold-based segmentation followed by a transformation from RGB to HSI colour space. Then, the lesion edges are identified using the Sobel operator and a second threshold is applied on the resulting image.	Soybean

Studies using visible features imaged with conventional RGB cameras have shown the ability for automated systems to recognise the presence of known plant disease using machine learning or deep learning models [25]. In this regard, a large number of studies have been reported in the literature that employed machine learning-based techniques for plant disease detection [26]. This approach can aid typical steps of image analysis including background removal and segmentation of the lesion tissue of the infected plants and discriminative feature extraction, which are fundamentals to determine the applicability of a machine learning model whose detection and severity estimation are generally based on [27]. However, these plant disease severity estimation methods are not fully automatic because they depend heavily on series of image-processing techniques, such as the threshold-based segmentation of the lesion area and hand-engineered features extraction [18]. Deep learning algorithms, such as models based on convolutional neural network (CNN), allow to automatically extract the features directly from the input images by-passing the background removal, segmentation, and discriminative feature extraction steps as well as providing more accurate results compared with traditional methods [28]. These approaches are remarkably powerful for solving classification problems but some other problems can not be represented in this form (i.e., fine-grained disease severity estimation). The major drawback is the need for a large set of data to train the models: an accurate generalised prediction (classification among different diseases) requires a large number of diseased and healthy plant images verified by expert plant pathologists. Furthermore, deep learning models rely on large neural networks that typically require an expensive training due to complex data models (possibly aggravated by data augmentation techniques) and the strategies learnt by deep learning may be more superficial than they appear [29]. Indeed, the fine-grained disease severity estimation is much more challenging, as there exist large intraclass similarity and small interclass variance [30].

The proposed model relies on a highly efficient and noise-rejecting positive non-linear dynamical system that makes use of an iterative colour discrepancy analysis technique to estimate the severity of pathogenic diseases and the proportion of symptomatic leaf area regardless of disease type. The main advantages of such an approach are:

1. the proposed system does not require any training to automatically discover the discriminative features for fine-grained disease severity estimation;
2. the model is robust even when only low-resolution and noisy images are available: the impact of noise (e.g., signal independent and uncorrelated noise) is effectively reduced and does not affect the final result;
3. the algorithm is able to detect symptoms belonging to previously unseen conditions, therefore it can potentially be applied to automated surveying systems.

The rest of this paper is organised as follows. In the Materials and methods section, we provide a detailed description of the mathematical model along with its properties, with particular attention to the transient behaviour and the convergence/divergence of the system. Then, the experimental results are reported and discussed in the Results section, with particular regard to the dataset used in the experiments and the experimental setup, parameter tuning, performance assessment, noise-rejection property, and computational efficiency of the algorithm. Finally, conclusions are drawn in the last section.

## Materials and methods

**Fig 1:**
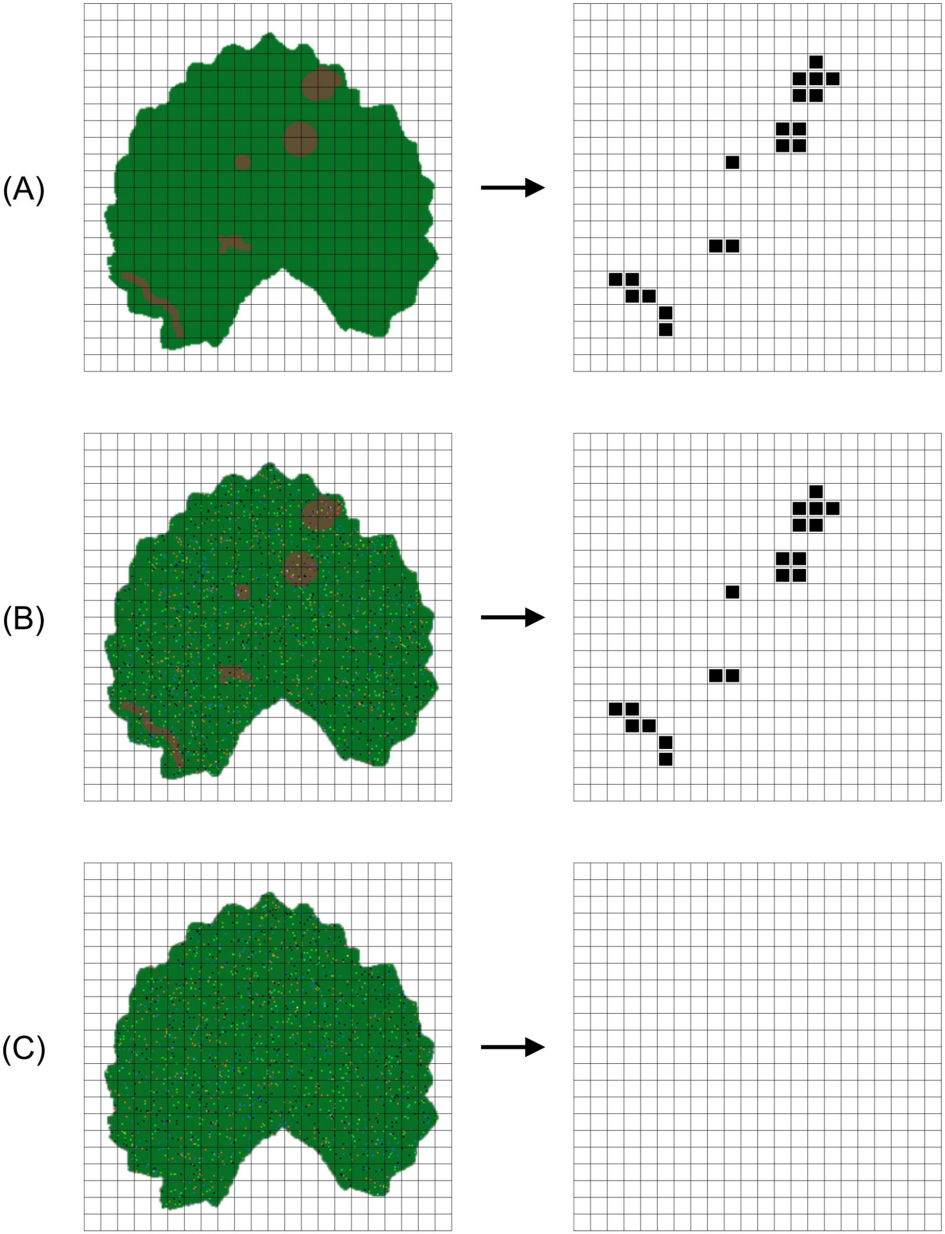
Desired output in different situations: The first column represents the initial (artificially created) image, whilst the second one represents the desired output matrix (active pixels in black). A, B: examples of correct detection when the input is a leaf image with disease symptoms upon pathogen infection (the second example considers a noisy input image). C: example of no detection when the input is a leaf image affected by impulse noise in healthy condition.

The idea behind the algorithm is to apply an iterative refinement technique based on the analysis of colour discrepancy between the points within the leaf area and a target colour that represents the symptomic areas, if any. Hence, the proposed dynamical system must behave as illustrated in Fig 1.

1. In the first example, a leaf image with disease symptoms upon pathogen infection has been provided as input. The output consists of a matrix with some sets of active pixels (by convention an active point has been represented in black, whilst a non-active point has been represented in white) representing the diseased regions of the leaf. Actually, almost all of the active points in the output matrix $Y=\tilde{X}$ should be superimposable to the visible symptoms presented in X.
2. The second example follows the same behaviour presented in the previous example with the only exception of the input image, which has been corrupted by adding a random impulse noise with probability *p* = 10%. The resulting output matrix $Y=\tilde{X}$ should be similar to that of the previous example (i.e., the noise does not affect the accuracy of the disease severity estimation).
3. In the third example, a leaf affected by impulse noise in healty condition has been provided as input. In this case, the output matrix $Y=\tilde{X}$ should be almost empty.

### Mathematical model

Positive dynamical systems are an important class of systems that arise naturally in many fields of science where the state-variables represent quantities that can only be positive (or at least non-negative) in value at all times. The explicit definition of a positive system is that its state and output are always non-negative for any non-negative initial state and any non-negative input. This non-negative restriction on system variables provides some remarkable outcomes that are available only for positive dynamical systems [31]. This section aimed at highlighting the main idea of the proposed system-theoretic approach for automatic disease severity estimation, which relies on a recursive algorithm based on a positive non-linear dynamical system whose evolution depends on the input tensor representing the leaf image to be analysed. Given an input RGB image $X\in R_{+}^{n\times n\times 3}$, it may be convenient adopting an operator of the form

$$\begin{array}{c} \tilde{X}=\Phi (X), \end{array}$$ (1)

which yields a Boolean matrix $\tilde{X}\in {\left\{0,1\right\}}^{n\times n}$ representing the affected area of the leaf image. Hence, the operator Φ must be chosen to assess the presence of a specific disease in the initial template image. To simply explain the idea, consider a simple (i.e., non dynamic) thresholding function. A simple option to seek the presence of visible signs and symptoms of the infectious disease is to consider a thresholding-based segmentation method in order to approximate the area of the diseased leaf on the basis of the different intensities or colours in the image:

$$\begin{array}{c} \tilde{X}=\{\begin{array}{cc} 1 & \text{if}\;∥t_{L}∥\leq ∥X_{i,j,:}∥\leq ∥t_{H}∥,\;t\in R_{+}^{3}\\ 0 & \text{otherwise} \end{array} \end{array}$$ (2)

where the threshold value is calculated according to a specific function. However, the approach based on this choice for the operator in Eq (1) is clearly not noise-rejecting, since noise inhibits the localisation of the threshold value. Indeed, to avoid a misleading diagnosis, noisy pixels that are present on the leaf region of the image should not provide a positive contribution to the severity estimation. Thus, it has been pursued an approach that rejects noise under the following assumption: the disease severity due to “isolated spots” is not as significant as that of cluster of points, whether they are wide “stripes” or “island”, even if the number of isolated spots is very high.

We assume that the leaf image provided as input, has already undergone a preliminary processing and that therefore contains the extracted region of interest. Thus, the behaviour illustrated in Fig 1 can be achieved by means of the proposed method which, in view of its iterative nature, it only requires to emphasise the presence of cluster points in diseased regions that differ in colour from those present in healthy regions. Hence, let **N**_*i*,*j*_ be the square neighbourhood of the generic point (*i*, *j*) within a “radius” *δ* of integer amplitude grater than zero

$$\begin{array}{c} N_{i,j}=\{h,l:∥h-i∥\leq \delta ,\;∥l-j∥\leq \delta ,\;h,l\in Z\} \end{array}$$ (3)

then, given a varying (i.e., tunable) tolerance $\xi \in R_{++}$ such that *ξ* < 1, the criterion used to determine whether a point $x\in R_{+}^{3}$ is subject to the *pathological condition* is defined as follows

$$\begin{array}{c} \nu =∥\frac{\bar{x}}{∥\bar{x}∥}-\frac{{\bar{x}}_{d}}{∥{\bar{x}}_{d}∥}∥\leq \xi \end{array}$$ (4)

where $\bar{x}$ represents the average of the RGB component vectors in the neighbourhood **N**_*i*,*j*_ of the observed point (*i*, *j*) at time instant *k*

$$\begin{array}{c} \bar{x}=\frac{1}{n(N)}\sum_{h,l\in N_{i,j}}X_{h,l,:}\left(k\right) \end{array}$$ (5)

whilst ${\bar{x}}_{d}\in R_{+}^{3}$ identifies the average colour components of the pathological condition.

Once this condition is met, the image is processed according to the updating equation

$$\begin{array}{c} X_{i,j,:}\left(k+1\right)=X_{i,j,:}\left(k\right)+\tau \rho_{i,j}({\bar{x}}_{d}-X_{i,j,:}\left(k\right)) \end{array}$$ (6)

where $X_{i,j,:}\left(k\right)\in R_{+}^{3}\;\forall k\in \left\{0,1,\ldots ,K-1\right\}$ represents the RGB vector of the observed point at the specific time instant *k*, the sampling time is defined by *τ*, and *ρ* governs the speed of convergence. Precisely, the function *ρ* represents a measure of how quickly the system can reach the steady-state condition and depends on the colour discrepancy between the vectors **x** and ${\bar{x}}_{d}$ (i.e., how far the observed point is from the *pathological condition*) which is defined through the following expression:

$$\begin{array}{c} \nu =∥\frac{x}{∥x∥}-\frac{{\bar{x}}_{d}}{∥{\bar{x}}_{d}∥}∥. \end{array}$$ (7)

Thus, given the measure of discrepancy *ν*, it is possible to calculate the speed of convergence *ρ* as follows:

$$\begin{array}{c} \rho =\frac{1}{1+\alpha \nu^{p}} \end{array}$$ (8)

where $(\alpha ,p)\in R_{++}$ are two coefficients to be set appropriately in order to meet the desired rate of convergence, as described in the Parameter tuning section.

At the final step, to achieve a Boolean image as illustrated in Fig 1, all pixels that have been affected by changes are set to one (i.e., all the labeled pixels) whilst pixels that have not been modified in any way are set to zero. The saturation function is defined as follows. Let $\tilde{X}$ be the resulting real-valued tensor having the same size as X, then the piecewise-defined function $\Theta :R^{3}\to \{0,1\}$ is called saturation function and is defined as

$$\begin{array}{c} \Theta (x)=\{\begin{array}{cc} 1 & \text{if}\;x\;\text{has}\;\text{been}\;\text{modified,}\\ 0 & \text{otherwise.} \end{array} \end{array}$$ (9)

Thus, through the function defined above, it is possible to generate the Boolean matrix $\tilde{X}$ of dimensions *n* × *n*, by means of the following computation for all *i*, *j*

$$\begin{array}{c} {\tilde{X}}_{i,j}=\Theta (X_{i,j,:}). \end{array}$$ (10)

To estimate the disease severity, we consider the area (relative or absolute) of the sampling unit (leaf) showing symptoms of disease expressed as a percentage or proportion [3, 32] of affected leaf area. The final score is performed on the number of pixels with value 1 (active), which is compared to the total amount of points within the leaf area. Hence, denoting by Σ(**S**) and $\Sigma (\tilde{X})$ the number of active points in the respectively matrices, the disease severity is defined as

$$\begin{array}{c} E(X)=\frac{\Sigma (\tilde{X})}{\Sigma (S)}. \end{array}$$ (11)

**Fig 2:**
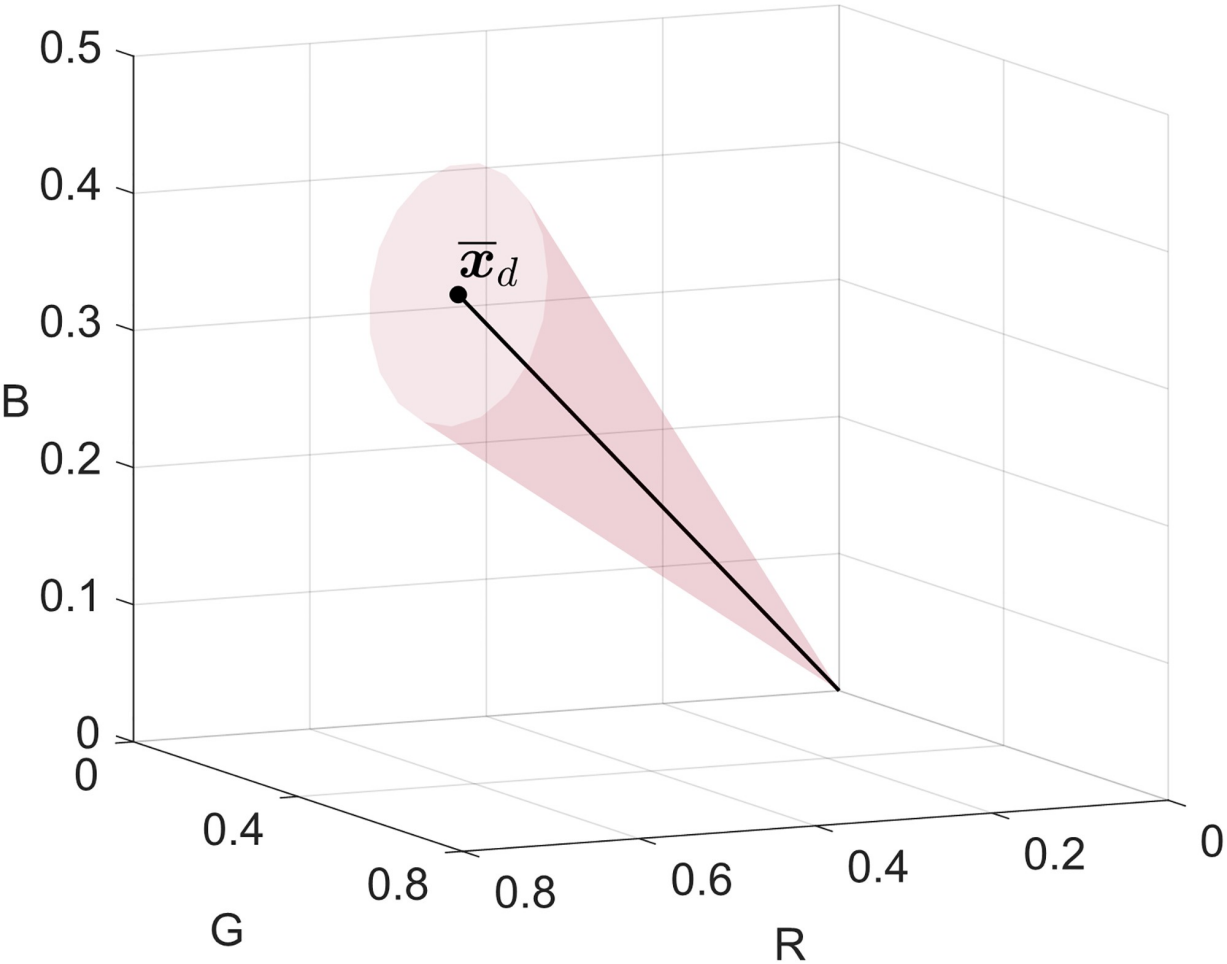
Maximal closed cone containing all accepted vectors in the normalised RGB colour space. The example shows a closed cone defined by the vector ${\bar{x}}_{d}$ and a tolerance *ξ* = 0.1.

The rationale of the core of the procedure is the following. Let X be the input RGB image and assume that it represents a diseased leaf, hence in correspondence of a visible symptom, the Eq (4) would be satisfied if the discrepancy between the normalised vectors **x** and ${\bar{x}}_{d}$ is less than or equal to the tolerance *ξ*. Fig 2, illustrates the maximal closed cone containing all accepted vectors in the normalised RGB colour space.

Actually, this is valid not only in correspondence of a visible symptom, but also in the nearest proximity of a visible symptom due to the mean filtering described in Eq (5), which reduces the amount of intensity variation between neighbouring points depending on the size of the square neighbourhood **N** defined through an integer radius *δ* > 0. Note that, even though the average of the RGB component vectors in the neighbourhood has been considered to test the pathological condition criterion, no point in the image has been replaced by this value, thus preserving image details. Then, assuming that the Eq (4) is satisfied, the speed of convergence can be computed by means of the Eqs 7 and 8. In particular, the Eq (7) measures the colour discrepancy between the observed point **x** (not to be confused with $\bar{x}$) and ${\bar{x}}_{d}$, which represents the colour of the *pathological condition*. Hence, through the Eq (8) it is possible to calculate the function *ρ* that affect the extent to which the observed three-dimensional vector **x** converges to that representing the *pathological condition* ${\bar{x}}_{d}$. Note that, in view of the two aforementioned equations, the greater the difference between the RGB vectors of the observed point and the *healthy condition*, the faster the convergence of the observed point to the *pathological condition*. Indeed, this behaviour is ensured by the dynamic Eq (6), which enables the vector **x** to asymptotically converge towards ${\bar{x}}_{d}$, usually in few iterations. Once the system has reached the steady-state condition (i.e., the condition in which the state variable is constant in spite of ongoing procedures that strive to change it) it is possible to estimate the disease severity through the Eqs (10) and (11).

**Fig 3:**
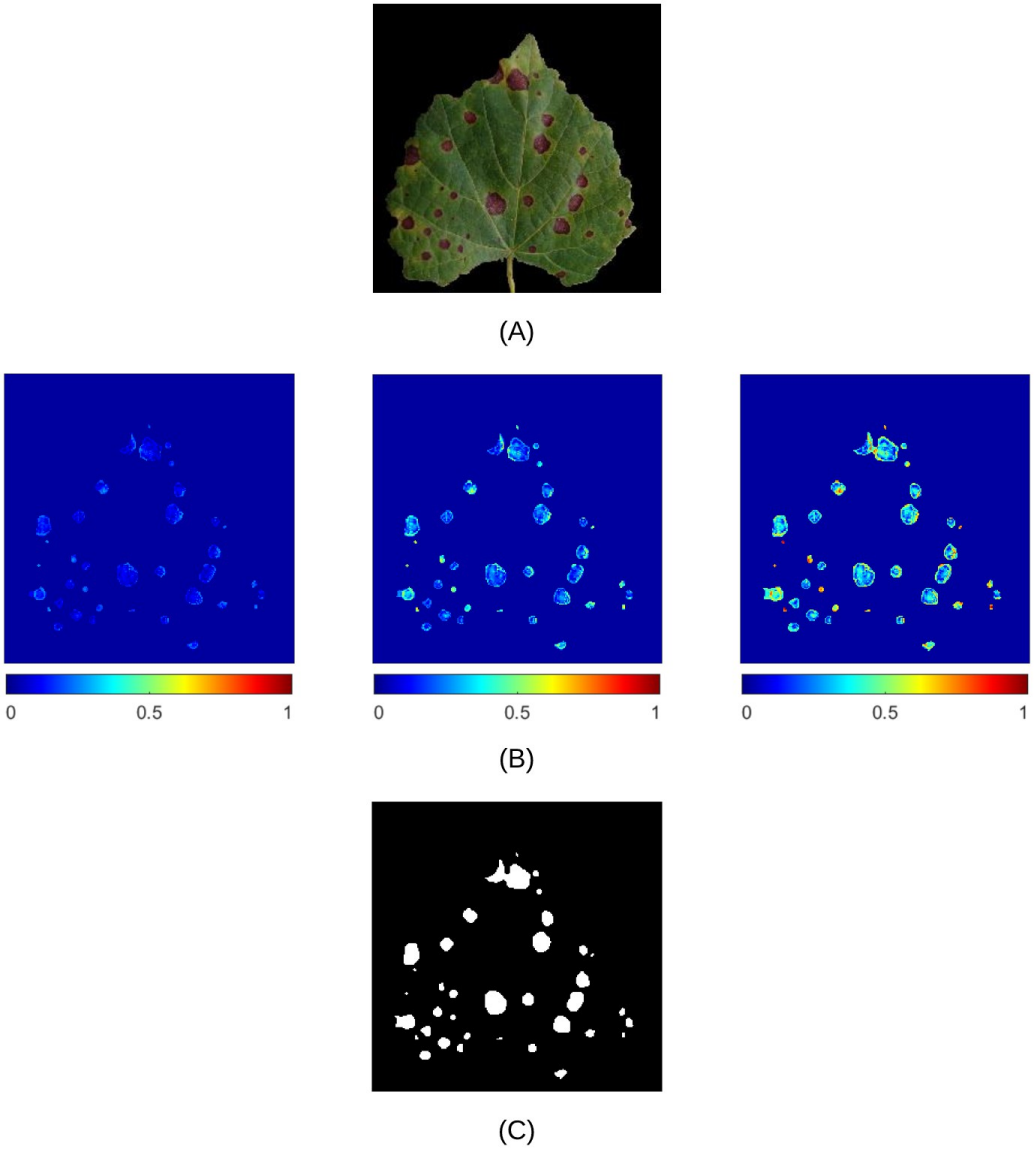
Dynamic algorithm behaviour. (A) Input image representing a grape leaf affected by *Black Rot* disease. (B) The resulting image from the application of the dynamic algorithm (representation of the pixels modified by the iterative procedure during the transient state until the steady-state condition has been reached). (C) Final result after binarisation.

To exemplify the algorithm behaviour, suppose that the input image X is that shown in Fig 3(A). Then, the result of the dynamic algorithm after the transformation to Boolean by means of the operator defined in Eq (1), which leads to the matrix $\tilde{X}$, is shown in Fig 3(C). In this example, the system has estimated a disease severity equal to $E(X)=\Sigma \left(\tilde{X}\right)/\Sigma \left(S\right)=0.0768$. The reader is invited to take a look at the video included in the additional material S1 Video to see the time evolution of the dynamic algorithm.

**Algorithm** Disease severity estimation

**Input**: leaf image X in RGB colour space.

**Parameters**: Number of steps *K*, positive real constants *ξ* < 1, *τ*, *α*, and *p*, vector ${\bar{x}}_{d}$, integer neighbourhood amplitude *δ* > 0 (which implies the size of the set **N**).

**Outputs**: Disease severity estimation E(X).

1. Set the initial condition X(0)≔X

2. **for** *k* = 0, *k* < *K*, *k* = *k* + 1

 **for each** point (*i*, *j*) belonging to the leaf area **do**

  compute the average of the RGB component vectors in the neighbourhood $N_{i,j}$ according to Eq (5)

$$\bar{x}=\frac{1}{n(N)}\sum_{h,l\in N_{i,j}}X_{h,l,:}\left(k\right)$$

  **if** the Criterion 4 $∥\frac{\bar{x}}{∥\bar{x}∥}-\frac{{\bar{x}}_{d}}{∥{\bar{x}}_{d}∥}∥\leq \xi$ is satisfied **then**

   compute the rate of convergence (speed) according to Eqs 7 and 8

$$\begin{array}{c} \nu =∥[\frac{x}{∥x∥}-\frac{{\bar{x}}_{d}}{∥{\bar{x}}_{d}∥}]∥\\ \rho =\frac{1}{1+\alpha \nu^{p}} \end{array}$$

   compute the updated value according to Eq (6)

$$X_{i,j,:}\left(k+1\right)=X_{i,j,:}\left(k\right)+\tau \;\rho_{i,j}({\bar{x}}_{d}-X_{i,j,:}\left(k\right))$$

  **end if**

 **end for**

**end for**

3. Convert the real-valued matrix to Boolean through the operator Θ as defined in Eq (9):

**for each** point (*i*, *j*) **do**

$${\tilde{X}}_{i,j}=\Theta (X_{i,j,:})$$

**end for**

4. Compute the disease severity as in Eq (11) $E(X)=\Sigma \left(\tilde{X}\right)/\Sigma \left(S\right)$, where **S** represents the Boolean matrix that identifies the leaf area of the initial RGB image X through pixels with value 1 (active).

#### Properties of the system

In this section we analyse the algorithm based on the recursive Eq (6), to better understand its behaviour. First of all, the positive parameters *α* and *p* in the procedure need to be tuned to define appropriately how the function *ρ* should behave. Let $v_{1},v_{2}\in R_{+}^{3}$ be two vectors to be compared, then it is possible to determine a metric defined on the normalised RGB colour space in order to find out the maximum differences along any coordinate dimension between two vectors (i.e., the maximum distance). Since the normalised RGB colour space is described by treating the component values as ordinary Cartesian coordinates in a Euclidean space that represents a cube of non-negative values such that $x_{i}\in R_{+}:x_{i}\leq 1\;\text{for}\;i=\left\{1,2,3\right\}$, it is convenient to consider a *L*^2^ norm. Hence, the real-valued function *ν* described in Eq (7) is positive and bounded above $\sqrt{3}$.

#### Property of convergence/divergence

Let us now consider just a point of a segmented RGB image $X\in R_{+}^{n\times n\times 3}$ representing a symptomatic leaf. Then the presence of diseased regions in the image give rise to a monotone system, as described next. Let us group in a vector $x\left(k\right)\in R^{3}$ the RGB component values *X*_*i*,*j*,:_ of the (diseased) point (*i*, *j*) in the image X.

Then, the system evolves as follows:

$$\begin{array}{c} x\left(k+1\right)=x\left(k\right)+\tau \rho ({\bar{x}}_{d}-x\left(k\right)) \end{array}$$ (12)

where

$$\rho =\frac{1}{1+\alpha \nu^{p}}$$

and ${\bar{x}}_{d}$ identifies the average colour components of the pathological condition. We further assume that the parameter *τ* representing the sampling time is chosen so as to guarantee that $1-\tau \rho \geq 0,\;\forall \;\rho \in R_{+}:\rho \leq 1$. Then, the vector **x** might be attracted towards the vector ${\bar{x}}_{d}$, and as such the dynamics of the system might lead to the filling of all diseased regions by acting on all the vectors that satisfy the criterion expressed in the Eq (4).

Let us consider the previous updating Eq (12) in the following form

$$\begin{array}{c} {}[\begin{array}{c} x_{1}\left(k+1\right)\\ x_{2}\left(k+1\right)\\ x_{3}\left(k+1\right) \end{array}]=\underset{\text{matrix}\;P}{\underset{︸}{\left[\begin{array}{ccc} 1-\tau \rho & 0 & 0\\ 0 & 1-\tau \rho & 0\\ 0 & 0 & 1-\tau \rho \end{array}\right]}}[\begin{array}{c} x_{1}\left(k\right)\\ x_{2}\left(k\right)\\ x_{3}\left(k\right) \end{array}]+\tau \rho [\begin{array}{c} {\bar{x}}_{d1}\\ {\bar{x}}_{d2}\\ {\bar{x}}_{d3} \end{array}]. \end{array}$$ (13)

Hence, it can be easily seen that the non-negative matrix **P** ≽ **O** appearing in the equation above is positive semidefinite and diagonal, which implies that the matrix is also symmetric (i.e., **P** = **P**^⊤^). Therefore, **P** is obviously a scalar matrix which can be viewed as a scalar multiple of an identity matrix. Note that multiplication by the identity matrix is equivalent to (scalar) multiplication by 1, and that multiplication by a scalar matrix (1 − *τρ*)**I** is equivalent to multiplication by the scalar (1 − *τρ*) [33].

**Fig 4:**
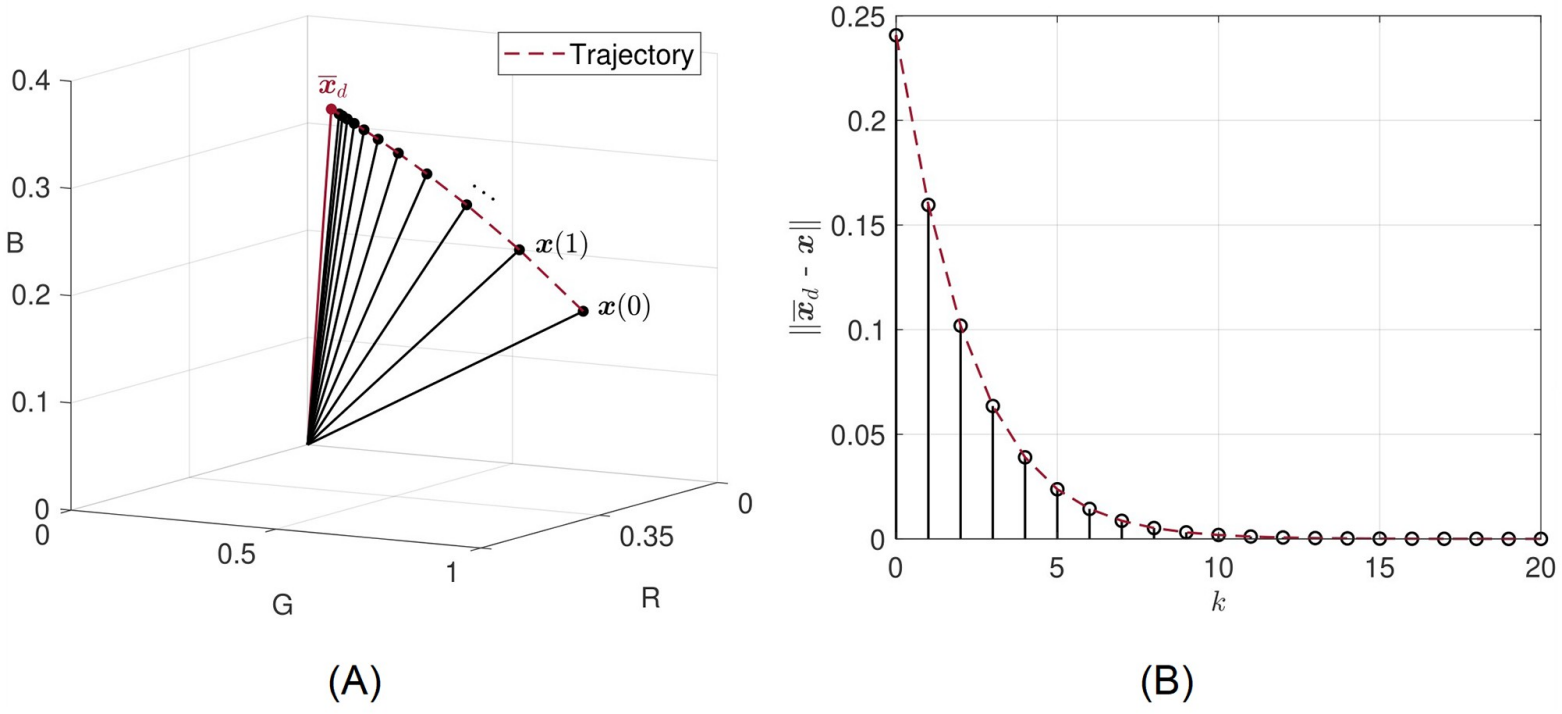
Transient behaviour. (A) A vector **x** (healthy) converges in norm to ${\bar{x}}_{d}$ (diseased). (B) Distance between the two vectors represented by the norm of their difference.

Moreover, since the matrix **P** has non-negative off-diagonal entries *P*_*i*,*j*_ ≥ 0 (∀*i*≠*j*), it is also a Metzler matrix. If the previous assumption holds, then the matrix **P** is called Schur stable, since all its eigenvalues lie inside the unit circle, or equivalently its spectral radius (i.e., the eigenvalue with maximum modulus) is non-negative, real, and equal to 1 − *τρ*. Furthermore, the term 1 − *τρ* serves to inhibit potential instability of the system because as **x** approaches ${\bar{x}}_{d}$, 1 − *τρ* approaches 0, ensuring thus a unique steady state that is globally asymptotically stable (monostability). Fig 4(A) illustrates the transient behaviour considering a point within the maximal closed cone defined by the vector ${\bar{x}}_{d}$ (i.e., the point satisfies the pathological condition in Eq (4)), whilst Fig 4(B) shows the distance between the two vectors represented by the norm of their difference.

## Results

Extensive experiments have been carried out to assess the performance and the effectiveness of the proposed algorithm, which are described in this section with particular regard to the dataset used in the experiments and the experimental setup, parameter tuning, performance assessment, noise-rejection property, and computational efficiency of the algorithm.

### Dataset and experimental setup

**Fig 5:**
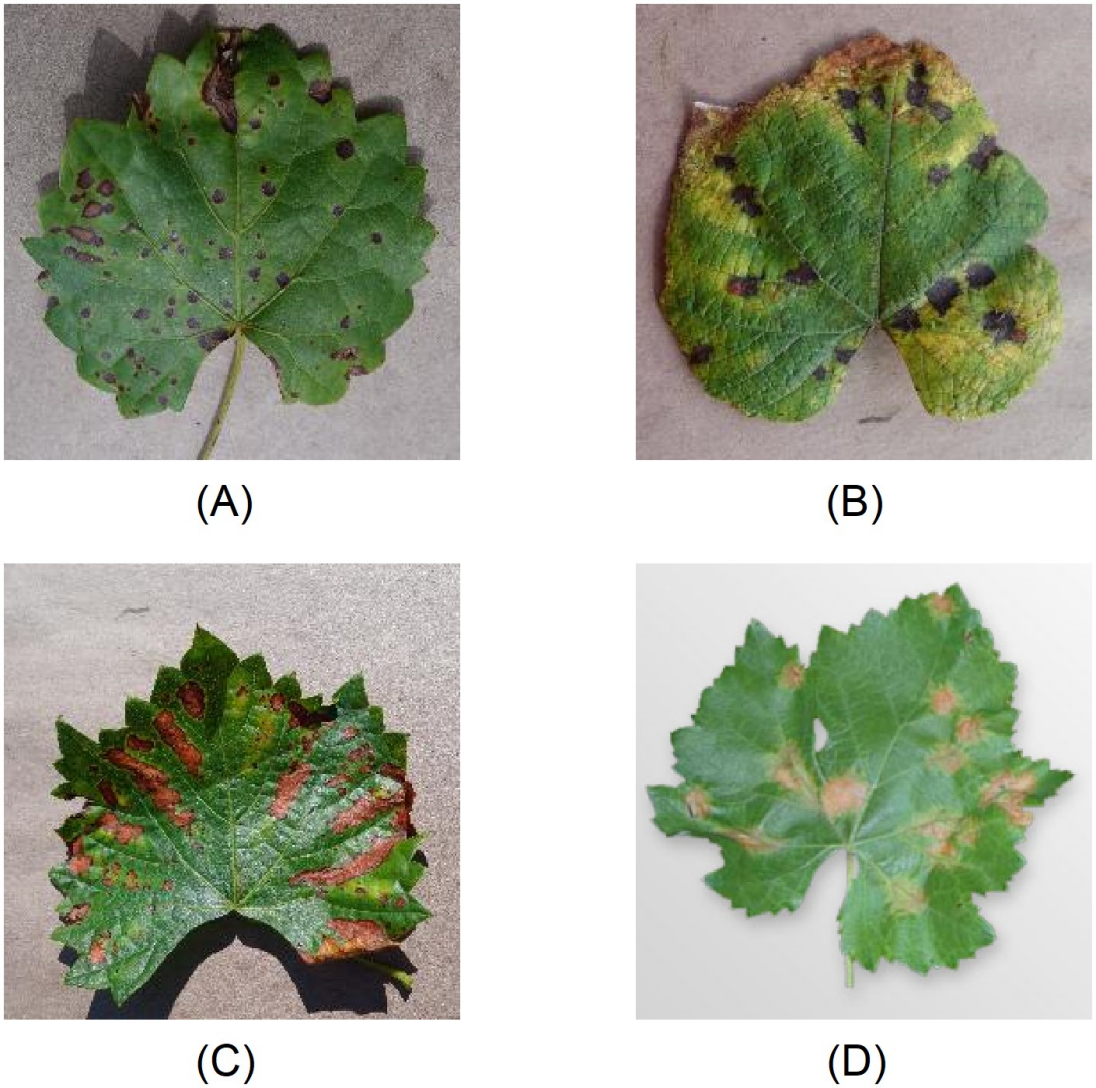
Examples of lesions on grape leaf caused by various infectious diseases. (A) Black rot (Guignardia bidwellii), (B) Leaf blight (Pseudocercospora vitis), (C) Esca (Phaeomoniella spp.), and (D) Downy mildew (Plasmopara viticola).

The dataset used to assess the performance of the proposed system, is based on the unmodified colour version of grape, peach, and apple leaf images in the PlantVillage dataset [34], which is worldwide shared for research purposes. Precisely, it consists of images of single leaves removed from their plants with inoculated or naturally occurring disease. The dataset used for evaluation purposes is composed of: (i) 2541 apple leaf images divided in 1645 healthy leaves and 896 leaves affected by various pathogenic diseases, (ii) 2657 peach leaf images of which 2297 leaves belong to the diseased class and the rest to the healthy class, (iii) 4063 grape leaf images of which 3640 are diseased leaves exhibiting three different conditions and 423 are healthy leaves, and (iv) 2152 potato leaf images of which 2000 samples present disease symptoms upon two different infectious pathogens whilst the rest are healthy samples. The conditions have been classified by expert plant pathologists by means of standard phenotyping approaches, therefore, only expertly identified leaves are present in the dataset. Leaf images have been captured through a twenty-megapixel camera (Sony DSC—Rx100/13 20.2 Mpx) using the automatic mode and collecting from four to seven different orientations to compensate for directional lighting variation. Indeed, all the images have been taken outside under nautral light in several different conditions (e.g., sunny, mostly/partly sunny, cloudy, and mostly/partly cloudy). The version of the dataset used in this study has been scaled down to 256 × 256 pixels and rotations of the same leaf have been removed [35]. Table 2 summarises the set of images used to test the system in disease detection configuration, whilst Fig 5 illustrates some sample images of lesions on grape leaf caused by various diseases used in the experiments (for more examples, see S1 File).

**Table 2:** List of crops and their disease status used in the experiments.

Culture	Diseased	Healthy
**Apple** (3172)	**Pathogen: fungi** *Gymnosporangium juniperi-virginianae* (276) *Venturia insequalis* (630) *Botryospaeria obtuse* (621)	(1645)
**Peach** (2657)	**Pathogen: bacteria** *Xanthomonas campestris* (2291)	(360)
**Grape** (4063)	**Pathogen: fungi** *Guignardia bidwellii* (1180) *Phaeomoniella spp*. (1384) *Pseudocercospora vitis* (1076)	(423)
**Potato** (2152)	**Pathogen: fungi** *Alternaria solani* (1000) **Pathogen: mould** *Phytophthora Infestans* (1000)	(152)

Hence, every single sample in the dataset as defined in Table 2 has undergone the test procedure of the system which calculate the percentage of infection over the leaf surface as in the Eq (11). Finally, the decision with respect to the estimated severity of the disease is as follows. Given a set of disease severity estimations **x**, the system has to determine if each element of the set belongs to the healthy group or not. Formally, the classification problem consists of determining if a disease severity estimation *x*_*i*_ belongs to the class of the null hypothesis *H*_0_ or to the alternative hypothesis *H*_1_:

$$\begin{array}{c} \left(x_{i},\psi \right)=\{\begin{array}{cc} H_{0} & \text{if}\;x_{i}>\psi ,\\ H_{1} & \text{otherwise.} \end{array} \end{array}$$ (14)

Precisely, given a threshold *ψ*, all disease severity estimation *x*_*i*_ lower (respectively, greater) than *ψ* lead to the rejection (acceptance) of *H*_0_ [36]. Therefore, whether the hypothesis is accepted or not, the test is prone to two kinds of error: (i) false acceptance rate (FAR), that represents the probability of accepting the null hypothesis when input *x*_*i*_ is below the threshold (type-I error), and (ii) false rejection rate (FRR), that represents the probability of rejecting the null hypothesis when input *x*_*i*_ is above the threshold (type-II error). The FAR and FRR are functions of the system threshold *ψ* and are closely related because the increase of the one implies the decrease of the other. Thus, it is not possible to decrease both these errors at the same time by varying the threshold value and therefore the system threshold *ψ* must be adjusted for the given application considering the trade-off between accuracy and false positives. The separation between the two classes, indicates the system ability to distinguish between diseased and healthy leaf samples. Indeed, the separation also provides a hint on the threshold point that maximises the variance between the two classes [31]. Once the threshold has been set, the reliability and validity of the scheme are determined by common measures that are used to evaluate the classification accuracy and effectiveness. In the presented experimental results, each disease has been treated separately leading thus to a dichotomous binary classification problem, where the labels are *P* (healthy) and *N* (diseased) and the predictions of a classifier are summarised in a 2 × 2 contingency table known as *confusion matrix* [37] (expanded in Table 3):

$$\begin{array}{c} M=[\begin{array}{cc} TP & FN\\ FP & TN \end{array}] \end{array}$$ (15)

which completely describes the outcome of the classification task. This contingency table may be expressed using raw counts of the number of records from class times each predicted label is associated with each actual class. As depicted in Eq (15), the matrix **M** reports:

- true positive (TP), the probability of correctly accepting the null hypothesis;
- true negative (TN), the probability of correctly rejecting the null hypothesis;
- false positive (FP), the probability of falsely rejecting the null hypothesis;
- false negative (FN), the probability of falsely accepting the null hypothesis.

**Table 3:** Example of confusion matrix for a dichotomous binary classification problem.

		**Predicted class**		
		**P**	**N**	**Total**
**Actual class**	**P**	*TP*	*FN* (Type-II error)	*TP* + *FN*
	**N**	*FP* (Type-I error)	*TN*	*FP* + *TN*
	**Total**	*TP* + *FP*	*FN* + *TN*	

Based on the entries in the confusion matrix, the total number of correct predictions carried out by the model is TP+TN, whilst the number of incorrect predictions is FP+FN [38]. Therefore, if

$$\begin{array}{c} M=[\begin{array}{cc} n^{+} & 0\\ 0 & n^{-} \end{array}] \end{array}$$ (16)

where obviously $n^{+}=TP+FN$ and $n^{-}=FP+TN$, then the classification has been perfectly done. Conversely, if the confusion matrix is as follows

$$\begin{array}{c} M=[\begin{array}{cc} 0 & n^{+}\\ n^{-} & 0 \end{array}] \end{array}$$ (17)

it represents the worst case (perfect misclassification).

Several measures have been defined to assess the quality of a prediction [39], aimed at conveying into a single figure the structure of **M**. The most used functions are briefly described as follows [36]. Precision, also known as positive predictive value (PPV) counts the true positives, how many samples are properly classified within the same cluster (closeness of the measurements to each other)

$$\begin{array}{c} PPV=\frac{TP}{TP+FP}. \end{array}$$ (18)

Sensitivity also known as recall or true positive rate (TPR) refers to the proportion of the samples properly classified as true positives out of the actual number of true positives

$$\begin{array}{c} TPR=\frac{TP}{TP+FN}. \end{array}$$ (19)

F-measure combines precision and recall in a single metric, indeed, it is the harmonic mean of precision and sensitivity and as a function of **M**, has the following form:

$$\begin{array}{c} F_{1}=2\frac{PPV\cdot TPR}{PPV+TPR}=\frac{TP}{TP+\frac{1}{2}\left(FN+FP\right)} \end{array}$$ (20)

where the worst case (*F*_1_ = 0) is achieved for *TP* = 0, whilst the best case (*F*_1_ = 1) is reached for *FN* = *FP* = 0.

Accuracy represents the ratio between the correctly predicted instances and all the instances in the dataset, whose range is between 0 (worst case) and 1 (best case):

$$\begin{array}{c} ACC=\frac{TP+TN}{TP+TN+FP+FN}. \end{array}$$ (21)

Matthews correlation coefficient is the measure of the quality of binary (two-class) classifications:

$$\begin{array}{c} MCC=\frac{TP\cdot TN-FP\cdot FN}{\sqrt{\left(TP+FP)(TP+FN)(TN+FP)(TN+FN\right)}} \end{array}$$ (22)

it is a correlation coefficient between the actual and predicted binary classifications and it returns a value in the range -1 (worst case) and 1 (best case).

### Parameter tuning

**Fig 6:**
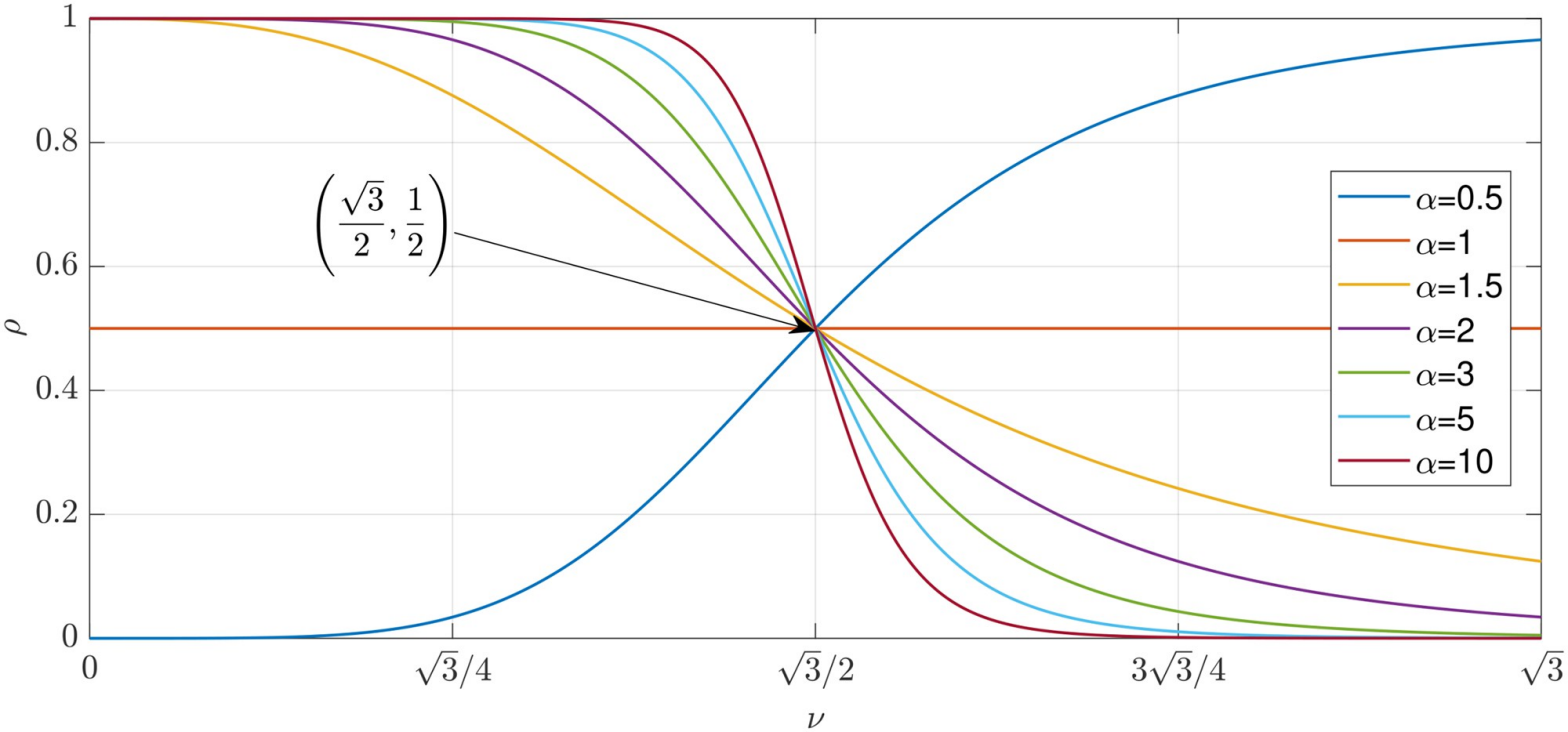
Example of several curves of the monotonic function *ρ* using several different parameters.

Since the proposed approach for matching is based on a non-linear parameter-dependent system, it is very important to set its internal parameters in order to maximise the system performance. The parametrs to be fixed are: *α*, *ρ*, and *ξ*. Firstly, we recall that the parameter *ρ* is calculated as in Eqs (7) and (8). We proved in the previous section that the parameter *ν* spans all the real values in the closed set $\left[0,\sqrt{3}\right]$. Thus, it is possible to test the behaviour of the function $\rho \in R_{+}:0\leq \rho \leq 1$ described in Eq (8) based on different values of the parameter pair $(\alpha ,p)\in R_{++}$, however, it should consider that greater the colour discrepancy between the vectors **x** and ${\bar{x}}_{d}$ (i.e., the observed point is far away from the *pathological condition*), smaller the value of *ρ*, and vice versa. Indeed, since this is a non-linear monotonic strictly positive function [40] and bounded from above by 1, the higher values should be achieved when the observed point is in proximity of diseased region. Conversely, when a deep mismatch between the two vectors is observed, the function should achieve a low (strictly positive) value, whilst in between the two cases the function should behave as an inverse S-shaped softening function with the point of inflection to be in the middle of the domain. Hence, the desired behaviour is ensured for *α* > 1. Let us consider the following function

$$\begin{array}{c} \rho =\frac{1}{1+\alpha \nu^{p}} \end{array}$$ (23)

where $\nu :R_{++}\to \left[0,\sqrt{3}\right]$, thus the problem is to find a parameter pair $(\alpha ,p)\in R_{++}$ such that when $\nu =\frac{\sqrt{3}}{2}$ we get $\rho =\frac{1}{2}$. Hence, by substitution we obtain

$$\begin{array}{c} \frac{1}{2}=\frac{1}{1+\alpha {\left(\frac{\sqrt{3}}{2}\right)}^{p}}\;\Rightarrow \;{\text{log}}_{\frac{\sqrt{3}}{2}}\alpha^{-1}=p \end{array}$$ (24)

and fixing the numerical value of the constant *α* > 1 therefore defines the other one in the proper way. In fact, considering the last equation, if 0 < *α* < 1 we get negative values for *p* which yields curves that rise rather than fall. The desired behaviour of the monotonic function is illustrated in Fig 6.

#### Estimation of the pathological condition vector

The vector ${\bar{x}}_{d}$ that better approximates the desired one, which would be used to test the pathological condition of a point (*i*, *j*) in proximity of ${\bar{x}}_{d}$, should be set considering the distributions of healthy and diseased regions of several digital leaf images and calculating common statistical parameters, e.g., mean, variance, and median. However, this approach may be time-consuming and unfeasible due to the lack of samples. From that, it follows that it is possible to find experimentally the most suitable value for each spot-based disease via grey histogram analysis. Indeed, the proposed approach relies on the identification of some diseased regions in order to compute the RGB vector we are looking for. Firstly, to accomplish this goal, we consider the average grey level from the grey-scale leaf image $G\in R^{n\times n}$. Hence, let $A\subset Z^{2}$ be the set of points belonging to the leaf area, then

$$\begin{array}{c} \bar{g}=\frac{1}{\text{n}(A)}\sum_{i,j\in A}G_{i,j}. \end{array}$$ (25)

Thus, the points that their gray level deviates from $\bar{g}$ by more than a threshold *t*, are assumed to be lesion spots. This is consistent with the process of disease appeared, which represents the evolution of pathological changes of the leaf from green to other colours. Note that this kind of approach is not suitable to find all the lesions on the leaf surface. Indeed, this aspect is not required at all, since we are only interested in finding a suitable RGB vector that represents well enough the pathological condition. Hence, the values of the vector components are not critical if we consider an RGB triple far enough away from the colour representing the healthy condition in direction of that of the pathological condition. For instance, selecting the marked points $\tilde{p}$ defined by their corresponding ones in the grey image **G** under the following condition:

$$\begin{array}{c} \tilde{p}=X_{i,j,:}\Leftrightarrow G_{i,j}\leq \bar{g}-t \end{array}$$ (26)

has worked satisfactorily for all the tested cases.

### Performance assessment

**Fig 7:**
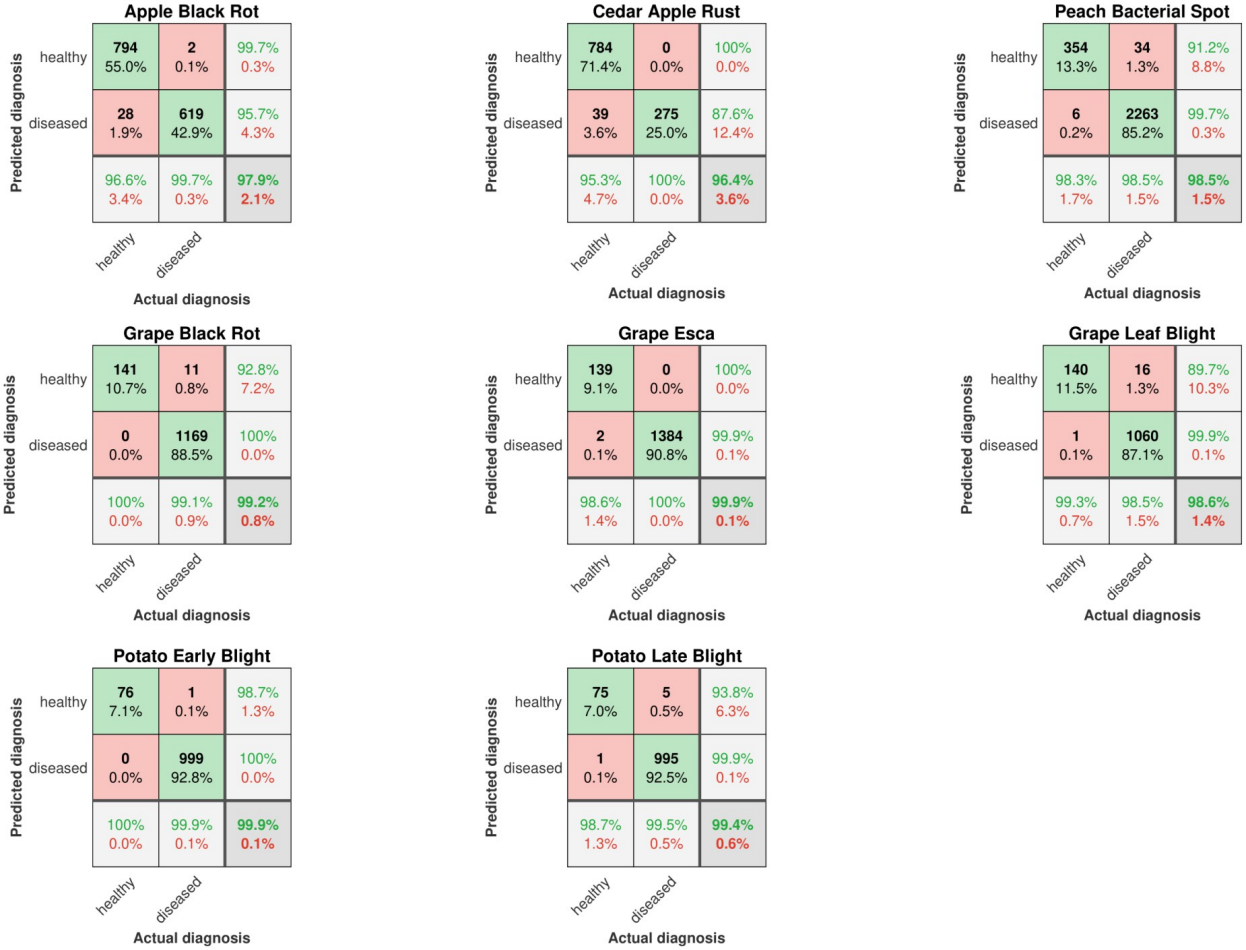
Confusion matrices for the proposed disease detection method.

As pointed out in [15], a fair comparison between different approaches would require an independent database that includes a wide selection of diseased crops with the related severity estimation properly labelled by expert pathologists, so as to be able to draw meaningful and comprehensive conclusions. This undertaking would be very demanding and should involve many people from different disciplines. The lack of such a database means that a truly direct comparison is not possible. However, meaningful conclusions can still be drawn if the analysis is performed in a more relative, less categorical context. Therefore, in order to investigate and to assess the performance of the proposed detection method that is agnostic to the type of disease, the dataset has been split into several different disease-healthy binary sets, each one considering only one specific disease. Fig 7 reports the confusion matrices for the proposed disease detection method. Note that testing the proposed system with several variations of the original dataset do not affect the results, since the proposed algorithm is invariant to rotation and translation.

**Fig 8:**
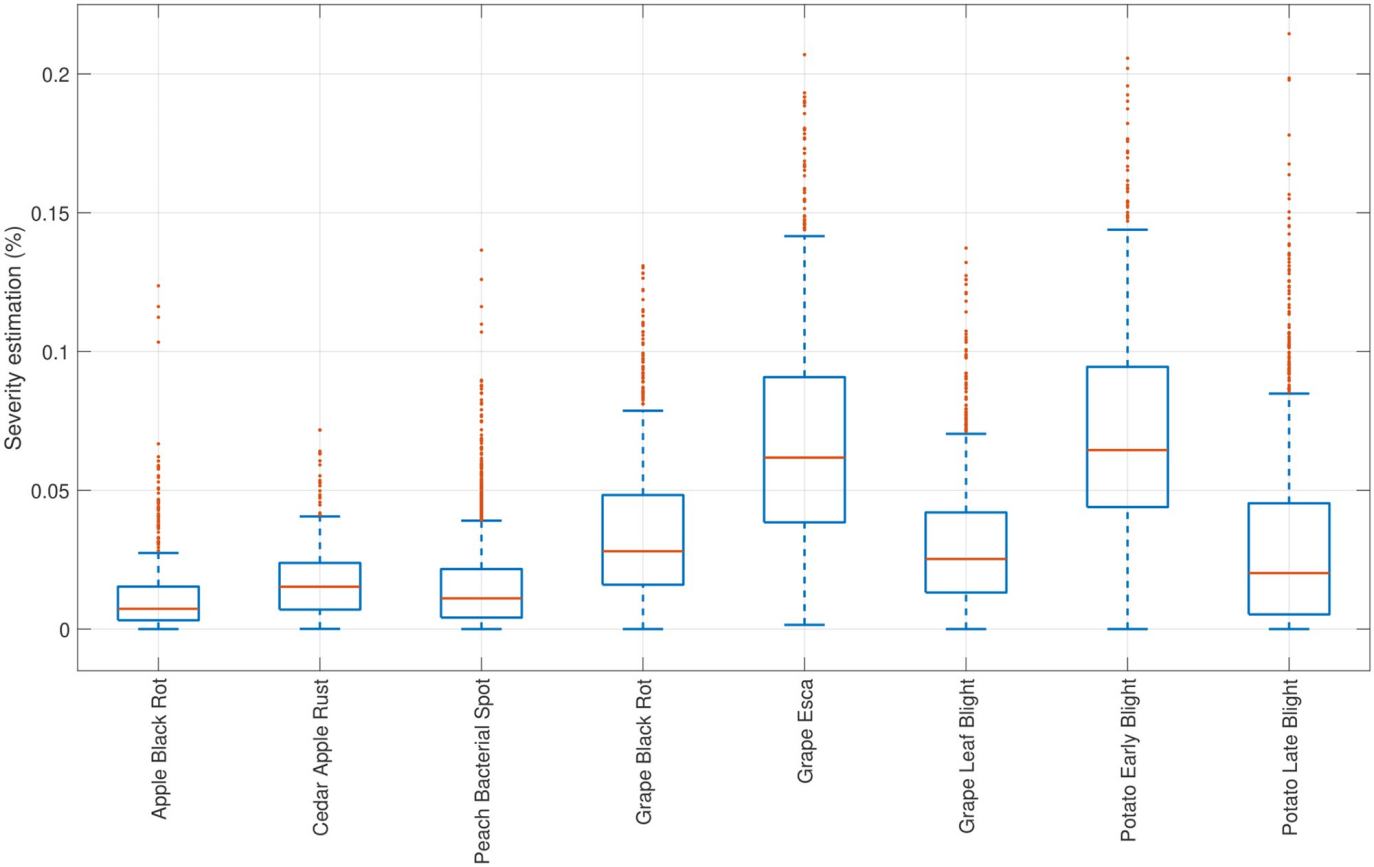
Disease severity statistical analysis through the boxplot of data results from each disease dataset.

Accuracy and F-score computed on confusion matrices have been (and still are) among the most popular adopted metrics in binary classification tasks. However, these statistical measures can show over-optimistic results, especially on imbalanced datasets as discussed in [39, 41]. Hence, among of all the parameters previously described, MCC is the only one that takes into account true and false positives and negatives, and is generally regarded as a balanced measure which can be used even if the classes are of very different sizes [42]. However, for the sake of completeness, we have summarised in Table 4 all the main measures. Actually, the results indicate that the algorithm performs incredibly well in localising symptomatic regions for disease detection achieving an average accuracy of 98.7% and thus demonstrating the effectiveness of the proposed system. Despite the inability to ground-truth boundaries due to subjectivity, the proposed algorithm has been consistently robust quantifying disease lesion from symptomatic leaf images. In Fig 8 it has been reported a statistical comparison between all the pathogenic diseases in terms of severity estimation, whilst the Table 4 lists the statistics of disease severity for each disease dataset.

**Table 4:** Summary of the experimental results in terms of disease detection and disease severity estimation for each disease dataset.

		Performance metrics					Disease severity statistics			
Culture	Disease	Accuracy	Precision	Recall	F_1_-score	MCC	Mean	Std dev	Max	Min
Apple	Black Rot	0.9792	0.9968	0.9567	0.9763	0.9585	0.0117	0.0142	0.1237	0.0000
	Cedar Apple Rust	0.9645	1.0000	0.8758	0.9338	0.9134	0.0183	0.0144	0.0717	0.0001
Peach	Bacterial Spot	0.9849	0.9852	0.9974	0.9912	0.9387	0.0156	0.0187	0.3479	0.0000
Grape	Black Rot	0.9917	0.9907	1.0000	0.9953	0.9586	0.0344	0.0237	0.1309	0.0000
	Esca	0.9987	1.0000	0.9986	0.9993	0.9922	0.0680	0.0371	0.2287	0.0015
	Leaf Blight	0.9860	0.9851	0.9991	0.9920	0.9364	0.0305	0.0231	0.1373	0.0000
Potato	Early Blight	0.9991	0.9990	1.0000	0.9995	0.9930	0.0706	0.0367	0.2410	0.0000
	Late Blight	0.9944	0.9950	0.9990	0.9970	0.9589	0.0322	0.0347	0.2145	0.0000
**Total average values**		0.9873	0.9940	0.9783	0.9856	0.9562	0.0352	0.0253	0.1870	0.0002

**Fig 9:**
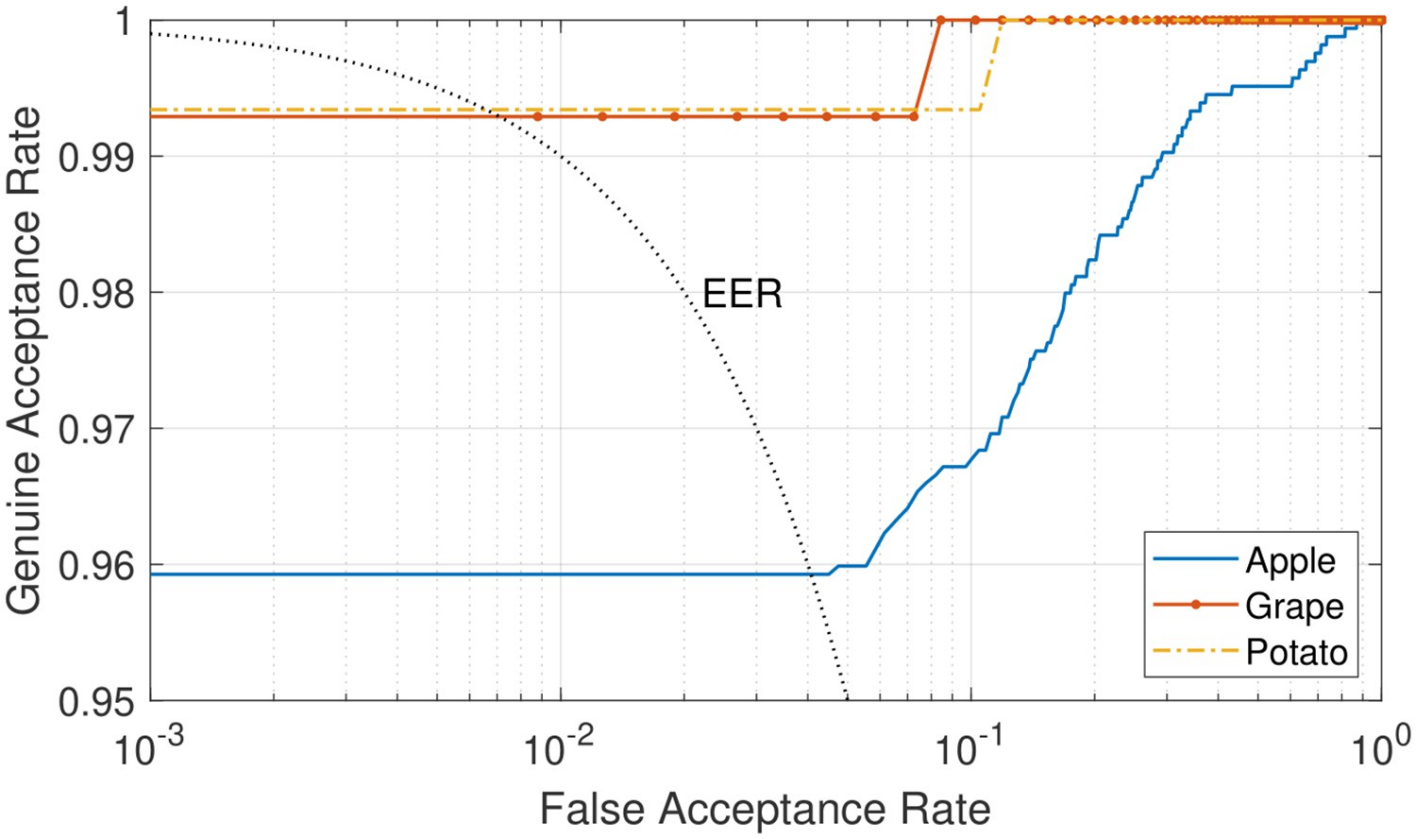
System performance analysis through receiver operating characteristic (ROC) curves obtained using the proposed approach for healthy and diseased classification over the apple, grape, and potato culture datasets.

Finally, we tested the system for healthy and diseased classification combining all diseases for each crop affected by more than one infectious pathogen (i.e., apple, grape, and potato crops). The experimental results are analysed in terms of receiver operating characteristic (ROC) and equal error rate (EER), which represent respectively the trade-off between FAR and FRR when the threshold varies and the intersection point for which rejection and acceptance errors are equal. In particular, the system achieved an EER equal to 0.0405, 0.0073, and 0.0068 for apple, grape, and potato cultures, respectively, whilst the ROC curves are obtained by plotting GAR = 1 − FRR against FAR, as illustrated in Fig 9.

#### Noise robustness

To conduct experiments on noisy leaf images and demonstrate the robustness of the dynamic algorithm with respect to noise, the system has been tested in noisy conditions. In particular, the impulse noise is a kind of noise which can have many different origins, often due to transmission errors, faulty memory locations, or timing errors in analog-to-digital conversion [43].

The impulse noise model has been defined through the following probability density function:

$$\begin{array}{c} p\left(z\right)=\{\begin{array}{cc} P_{a} & \text{for}\;z=a\\ P_{b} & \text{for}\;z=b\\ 0 & \text{otherwise.} \end{array} \end{array}$$ (27)

**Fig 10:**
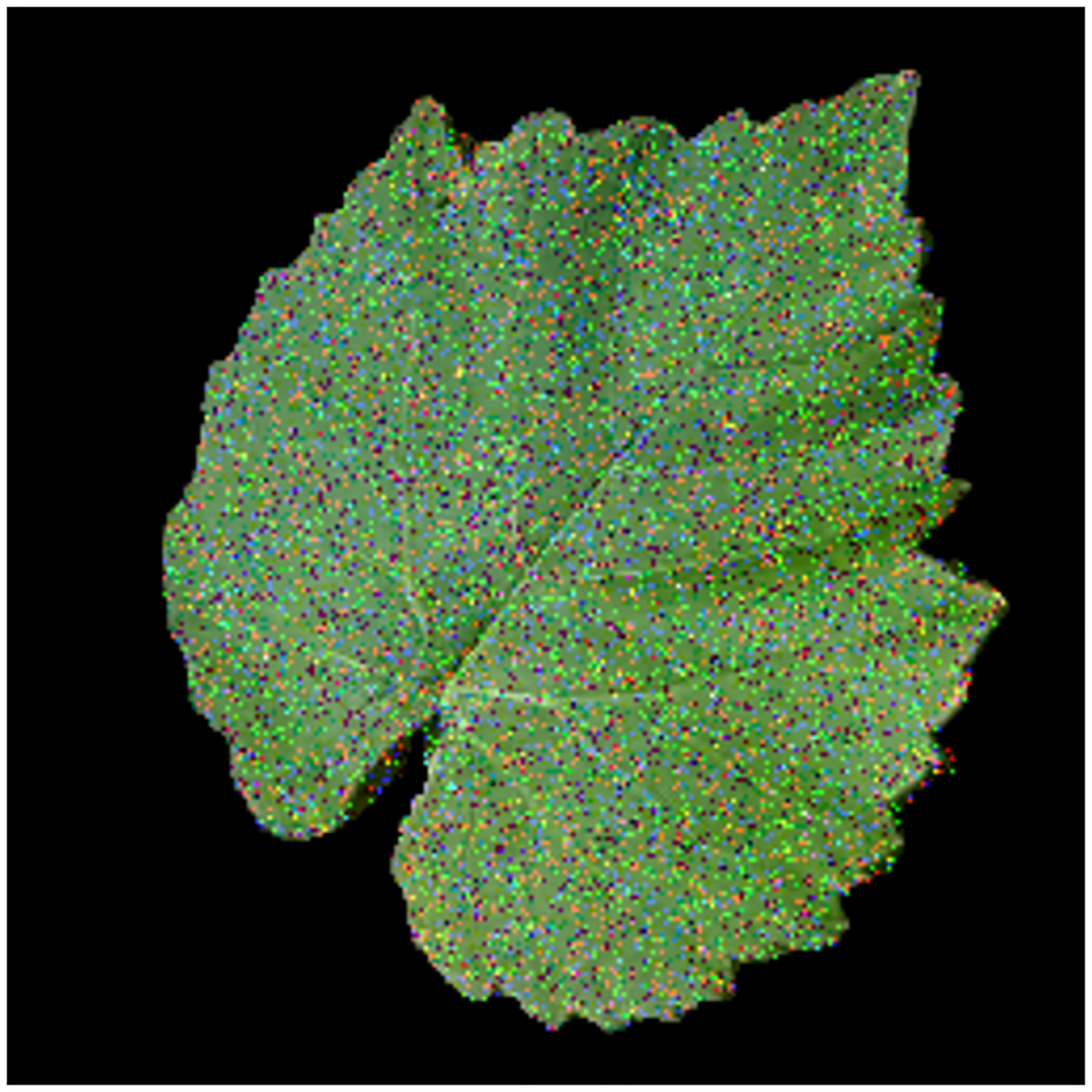
Example of healthy leaf image affected by impulse noise with a probability *p* = 15%.

If *b* > *a*, the intensity level *b* will appear as a bright spot in the image. Conversely, the intensity level *a* will appear as a dead spot. If either *P*_*a*_ or *P*_*b*_ is zero, the impulse noise is called unipolar. If neither probability is zero, and especially if they are approximately equal, impulse noise values will resemble salt-and-pepper grains randomly distributed over the image [44]. Therefore, in the case of the RGB colour space, such a noise is always independent, randomly distributed, and uncorrelated with respect to each colour component. We can distinguish two cases, (i) the first one arises when the image affected by the noise represents a leaf in pathological condition and (ii) the second one considers the case of an image with no diseased regions (healthy leaf). However, the second case is more challenging with respect to the first one, since the presence of noise in a healthy leaf image may lead to a type-I error (false positive) in the detection of the disease, whilst this is not a problem at all in the case of a symptomatic leaf image. Thus, the experiments to test the noise robustness of the system have been conducted considering only the dataset containing healthy leaves, an example of which is depicted in Fig 10. Table 5 reports the results of noise-rejection experiment and shows that the performance of the system is not that much degraded: even in presence of noise, the system is able to correctly detect a healthy leaf with an accuracy equal to 97.4%, 94.1%, and 91.5% for *p* = 5%, *p* = 10%, and *p* = 15%, respectively. Thus, the results presented in this section demonstrate the validity and effectiveness of the proposed system-theoretic approach for image-based infectious plant disease detection and severity estimation regardless of pathogenic disease type.

**Table 5:** Summary of the noise-rejection experiment results in terms of accuracy and error rate on the healty leaves of the whole dataset.

Noise probability	Performance metrics			
	Accuracy	TP	FP	Error rate
*p* = 5%	0.9740	412	11	0.0260
*p* = 10%	0.9409	398	25	0.0591
*p* = 15%	0.9149	387	36	0.0851

## Conclusion

In this paper, a novel system-theoretic approach for automatic image-based infectious plant disease detection and severity estimation has been investigated. The system relies on a highly efficient and noise-rejecting positive non-linear dynamical system that makes use of an iterative colour discrepancy analysis technique to estimate the severity of pathogenic diseases and the proportion of symptomatic leaf area regardless of disease type. In particular, the idea that characterises the algorithm is to apply an iterative refinement technique based on the analysis of colour discrepancy between the points within the leaf area and a target colour that represents the symptomic areas, if any. A noticeable advantage of such an approach is that the model does not require any training to automatically discover the discriminative features for fine-grained disease severity estimation. In addition, a peculiar property of the system relies in the robustness when dealing with low-resolution and noisy images. Indeed, an essential issue related to digital image processing is to effectively reduce noise from an image whilst keeping its features intact. The impact of noise (e.g., signal independent and uncorrelated noise) is effectively reduced and does not affect the final result allowing the proposed systems to ensure a high accuracy and reliability. Moreover, the proposed experimental setup allowed to assess the system ability to generalise symptoms detection beyond any previously seen conditions achieving excellent results, even in adverse conditions (e.g., in presence of significant noise). This kind of flexibility is not present in automatic methods, which usually have to deal with the problem based only on the training data and in the fine-tuning of area measurement. A further advantage of this algorithm compared to those in the literature is that its implementation is very simple and straightforward as it is entirely based on a simple non-linear mathematical model and some ad-hoc rules. This also makes it suitable for implementation in resource-constrained devices. Finally, even though this study is a first step towards a fully automatic diagnosis of plant disease severity, the model has proven to be highly accurate and robust and the experimental results are very promising also allowing the potential to provide new applications for infectious disease screening. Indeed, in addition to monitoring epidemics, an accurate assessment of infectious diseases severity is also critical for studies of genetic resistance, germplasm evaluation, and breeding.

## Supporting information

S1 File Diseased grape leaf image samples. Several examples of grape leaf image affected by different pathogenic diseases.
(PDF)

S1 Video Time evolution of the dynamical system. The video shows the dynamic simulation results in terms of transient and steady-state responses on a healthy leaf and a leaf in presence of pathological conditions.
(MP4)
