# Supplementary material for: A system-theoretic approach for image-based infectious plant disease severity estimation
Source: PLoS One. 2022 Jul 26;17(7):e0272002. doi: 10.1371/journal.pone.0272002 (PMC9321435; doi:10.1371/journal.pone.0272002)

A system-theoretic approach for image-based infectious plant disease severity estimation

David Palma, Franco Blanchini, and Pier Luca Montessoro

S1 File. Diseased grape leaf image samples. Several examples of grape leaf image affected by different pathogenic diseases.

Black rot	Esca	Leaf blight	Healthy

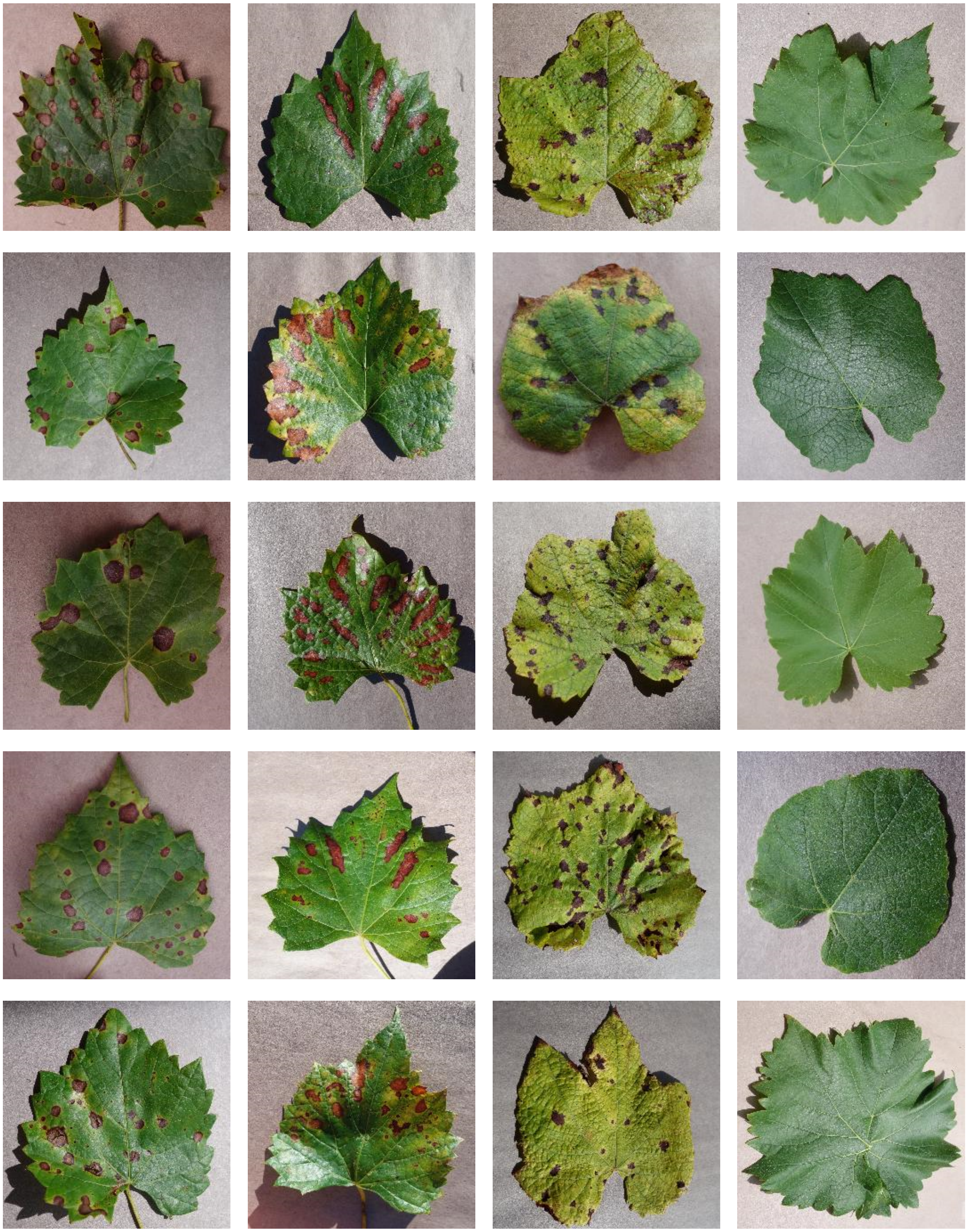

Supplement: S1 File — Several examples of grape leaf image affected by different pathogenic diseases. (PDF) [file pone.0272002.s001.pdf]
